# Play Therapy to Control Pain and Suffering in Pediatric Oncology

**DOI:** 10.3389/fped.2016.00132

**Published:** 2016-12-08

**Authors:** Dorella Scarponi, Andrea Pession

**Affiliations:** ^1^Unità Operativa Pediatria, Azienda Ospedaliero-Universitaria, Policlinico S. Orsola-Malpighi, Bologna, Italia

**Keywords:** oncology, pediatrics, play therapy, psychotherapy, family

## Pediatric Pain in Oncology

“*Pain can be defined as an unpleasant sensation and an emotional experience endowed of a negative affective tone, associated with a potential or real tissue damage and, anyway, described in relation to this damage*” (1). In other words, pain is perceived as such by each individual. Whatever its origin, it provides defense mechanisms and protective reactions, which modify the psychological trauma risk management (TRiM): mood alteration, anxiety, depression, fear, and rage, as well as the somatic TRiM: antalgic posture, facial expression alteration, activation of the autonomous nervous system, tachycardia, nausea, vomiting, and sweating (2). For these reasons, “total pain” is now recognized as a complex of physical and emotional suffering (fear, anxiety, depression, and rage), linked to cultural, spiritual, and social factors.

In pediatric patients, pain frequently occurs during diagnostic and therapeutic procedures. All children have an irrational fear of these maneuvers, particularly punctures, which are experienced as a physical intrusion in the body, through the barrier of the skin. Anxiety and tension worsen the pain, amplifying the perception, and voiding their psychophysical resources. Distraction and cognitive behavioral techniques aim to reduce these correlated factors, “shifting” the cognitive and emotional attention onto something else, and controlling muscular contraction, nausea, vomiting, and migraines (3). Analytically oriented individual and group psychotherapy is a non-pharmacological technique used for controlling the perception of pain and for communicating the most intimate feelings related to dramatic illness events (4).

### Procedural Pain

Acute pain induced by a medical procedure is one of the most frequent experiences in pediatric oncology. The correct approach to adequately contain the experience of acute suffering, according to Italian guidelines (5), is to rely on the integration of pharmacological and non-pharmacological methods, incorporating a *global, individualized, and effective approach*, with the purpose of “removing” the pain and minimizing the related stress and fear. The psychosomatic integration of the treatments, which depends on the multidisciplinary work of the staff, requires the presence of a psychotherapist, who can provide support to the patients and their parents and facilitate the communication about the experience of pain and suffering (6). The emotional and rational comprehension of the phenomenon of pain usually enables patients and their parents to:

– become aware of the communicative style, within the family;
– understand the psychological and existential meaning of their own pain;
– control the mental aspects of the pain.

### Pain and the Experience of Pain

The treatment of cancer pain in children requires the knowledge of all the factors that contribute to define the painful experiences as a whole, i.e., the level of the children’s psychological and physical development, their cognitive and emotional abilities, their main personality traits, their past experiences, and their family context. These are all essential to evaluate and effectively treat the pain. Understanding the child’s “pain experience” helps us to identify the most effective pharmacological treatment and to create a good relationship between the doctor and the patient, the so-called therapeutic alliance (7).

Supported by an integrated approach, involving clinical and emotional aspects, many Italian hospital programs encourage the young patients to attend play therapy sessions, as an educative supportive instrument, in order to face the fear of invasive and painful medical procedures and to improve treatment compliance (8). Our ongoing experience, which considers *play therapy* as a specific psychological therapy, is centered on the group-analytic theory, which can also be used in hospitals, is based on these grounds (9).

## Play Therapy to Support Children in Hospital

Play therapy is an ideal opportunity where suffering children usually act out characters, share experiences, and discuss the fears of being patients. In the group room, there is a small table in the middle and an intimate circle of chairs. The group therapist is part of the circle. Playing with someone else, within a therapeutic relationship, in the hospital, becomes an occasion of encouragement to stage dramatic intimate stories related to pain and suffering, in a transition area, between reality and imagination. At the same time, patients can find explanations to their questions and alternative imaginative endings regarding their illnesses, thanks to the interaction with other people; thus, it is possible to change the perspective of pain, in order to evaluate the events and add new elements of hope. The therapeutic relationship, similar to the primary parental one, reintroduces a deep emotional climate that could be corrective, thus providing the opportunity to repeat certain steps of the child’s own life, not only as a direct repetition but also as a variation (10). The supportive and analytic factors of the group, namely, the confidential link between the patients and their therapist, the atmosphere of free-floating discussion, and the climate of easy permissiveness, all enhance the creation of a good matrix, as a secure base, where children can try new ways of facing the events (11). The creation of mutual storytelling, within the group-setting, encourages greater autonomy, different perspectives of the events that the patients are living, and a sense of hope, so that the children feel more able to adopt different strategies to deal with their illness. Playing is, for every child, an occasion to experience satisfaction, to play the role of the main character, to invert these roles, and to imagine new endings, through fantasy. Chronic diseases, in fact, reduce the ability to create and to invent, because the disease and the medical therapies usually introduce a series of lifestyle changes, which impose limitations on the children’s talents. The negative emotions linked to painful experiences confirm the idea of being completely under the power of the events, without any chance of controlling them (12); these therefore reduce the children’s trust and hope for being in charge of their lives. When the chronic disease is potentially mortal (for example, cancer), the feelings are complicated by psychic impotence and sorrow. Play therapy, in these particular contexts, becomes an important occasion to communicate and to reobtain some normality in their life (13).

### Sessions of Play Therapy

All patients are allowed to attend the daily session of group play therapy before and after the painful maneuvers, to guarantee the continuity of the psychological intervention. During a play therapy group, we can observe several modalities of games, namely,

– direct medical games: children take the role of the different operators and, by simulating the painful maneuver that they are going to face, are able to reduce the expectation anxiety. Often, the patient identifies himself with the physician or the nurse and, in doing so, expresses the need to control the events, submitting the others (dolls, puppets, or the psychologist) to the same treatments and maneuvers that give rise to so much fear and distress. Thanks to the repetition on the toy of their own condition, the child gives himself the possibility and freedom to overturn his passive attitude and to dominate the frustrating experience, in his own way;
– fantasy medical games: children who are scared by the interventions do not use false or real medical instruments, but shift to common objects, which are more familiar and less evocative, in order to feel less involved;
– indirect medical games: children manipulate real medical instruments, using them in a new way, to make them less scary and, as such, the situation less dramatic;
– drawing: children can express their emotions and give them a shape, through graphic-pictorial activities.

Being together, thanks to mirroring, the group activities promote socialization, which allows the children to share the same experiences, reduce the fear, and acquire new modalities to face the stress (14).

### Pain That Can Be Told

#### The Story of a Clinical Experience

The first semi-open group began with five patients, lasted for 12 meetings, and ended with the discharge of the patients from the Day Hospital regime by the Christmas holidays. The whole group was constantly affected by the liveliness of one 10-year-old boy who liked to act as a leader, recounting his adventures outside of the hospital. All the children listened to him mesmerized. L., one of the patients, did not tolerate being part of the group and dropped out after the first meeting. L. comes from a family who do not talk about L.’s illness and in which the only non-familial intervention is the teacher. F. and I. were quiet but paid close attention to the aforementioned leader’s stories. As the group continued, they all took a more active part in the drawing productions as well as in the verbal communication. When a child was called out (for a blood transfusion or for a medical examination) the remaining children tried to imagine the type of procedure that the child was undergoing. When there were only two or three children, communication was more intimate and the favorite subjects were pain and feelings of sadness. One time there were three patients, F., I., and C., all peers, and they started to talk about painful practices. On this day, C. had to undergo a lumbar puncture and had already taken a sedative. He started to speak slowly, but he wanted to play with us. He drew scary monsters and asked me to draw other monsters. He then attacked them and scribbled them out with a pen. He told us what he usually does to avoid the feeling of pain: he screams really loud so he cannot feel it. I., instead, asked him to concentrate on something else. F., on the contrary, does not cry anymore. When, during the group session, the mother of C. came inside to call him, he asked if it was really his turn. The others kept working while I told him that we will wait for him and we will save his place with his drawings. After the medical intervention, his mother came back inside again and said that C. had to tell me something. Lying on the bed with half-closed eyes, C. told me: “This time I did not cry!” This was the first time he did not cry. I answer that he was very brave. I suppose that, perhaps, he has been telling us so much about his experiences and in such a clear way that he did not have anything more to scream about, during the puncture. “That must be right … now I’ll sleep a while,” he replies. I tell the C.’s story to the small group. They are reassured by the fact that everything went well. During the last session of this group, there are three of us. We know that we are going to say “goodbye” before the Christmas holidays. In the penultimate meeting, F. wore a very nice green outfit, which the rest of the group noticed. On this occasion, I. left an incomplete drawing because he did not like it. F. asked him to complete it. There were some small houses, without roofs or windows. F., considering that he was going back home, was telling us about the time spent with his brother and his friends from school, and his desire to become a shepherd, when he grows up. I looked at him in an affectionate way. He could not explain his own desire to go back to school as he had so many problems with his sister in the class! Today, while F. is already sitting at the table, I. enters the room with his mother. I immediately notice that he is wearing a new outfit, not blue as usual, but green, a forest green. His mother tells me that he wanted to buy some clothes yesterday afternoon. Now, the two boys look a little alike, which I voice out loud. I. is happy, he feels special. Together, with F., they start to draw. I. tells us what makes him angry, continuing the conversation from the time before. F. has fun when he gets angry! Anyway, both children clearly express their feelings. The group lasts a little longer than usual. When I bring the session to a close, they are the last ones left on the fourth floor of the Hospital. We say “goodbye” with a hug. I take a last look at the tone of their skin, so different, and at the same forest green color of their clothes, etc. (15).

## Some Final Considerations

Cancer pain is a complex experience consisting of many different factors. The perception of pain makes its somatic component deeply complicated by psychosocial elements, which are able to modify the pain itself. Non-pharmacological techniques are particularly used in pediatrics to control the psychological aspects of pain that characterize the experience of suffering. The group play therapy is a specific technique that we use in the hospital not only to control the pain but also to give it a mutually psychological meaning. The analytically oriented group is, in effect, an elective frame where psychotherapeutic factors, as defined by Foulkes, find ways to incorporate patients into the group and encourage them to reject isolation and to look for new shared solutions.

## Author Contributions

All authors listed, have made substantial, direct and intellectual contribution to the work, and approved it for publication.

## Conflict of Interest Statement

The authors declare that the research was conducted in the absence of any commercial or financial relationships that could be construed as a potential conflict of interest. The reviewer and handling editor declared their shared affiliation, and the handling editor states that the process nevertheless met the standards of a fair and objective review.

## References

[1] World Health Organization (WHO). WHO Guidelines on the Pharmacological Treatment of Persisting Pain in Children with Medical Illnesses. Geneva: WHO Press (2012). Traduzione italiana Organizzazione Mondiale della Sanità (OMS) Linee guida dell’OMS sul trattamento farmacologico del dolore persistente nei bambini con patologie croniche gravi, Milano, Fondazione IRCCS, Istituto Nazionale dei Tumori (2014).

[2] Rajapakse D Liossi C Howard RF Presentation and management of chronic pain. Arch Dis Child (2014) 99:474–80. 10.1136/archdischild-2013-304207 24554056

[3] Finelli P Scarponi D Pession A La clownerie non è una scienza. Milano: Casa Editrice Ambrosiana (2016).

[4] Scarponi D Dolore procedurale e sofferenza in incoematologia pediatrica. La giocoterapia come supporto psicologico. (Vol. 9). Roma: Giornale Italiano di PsicoOncologia (2008). N. 1/7.

[5] Benini F Il dolore nel bambino. Quaderni acp (2010) 17(2):70–3.

[6] Scarponi D Bevilacqua A Expériences d’animation de groupes pour les parents d’enfants malades du cancer. Une lecture chronologique de trois expériences d’animation de groupes pour les parents des petits patients en oncologie. Psycho Oncol (2013) 7:146–9. 10.1007/s11839-013-0425-3

[7] Jankovic M Van Dongen-Melman JE Vasilatou-Kosmidis H Jenney ME. Improving the quality of life for children with cancer. European School of Oncology Advisory Group. Tumori (1999) 85(4):273–9. 10587031 10.1177/030089169908500412

[8] Scarponi D Puglisi I Pession A Baroncini S Pain free hospital: an integrated approach to the oncological paediatric pain. 16th International Conference on Health Promoting Hospitals and Health Services Berlino: Conference Abstract Handbook (2008). p. 51–3.

[9] Mazza S Scarponi D Il gruppo di lavoro ad orientamento analitico per i bambini. XVI Congresso Nazionale SIPPS. Genova 20-21. Minerva Pediatr (2004) 56(Suppl 2 n 6):58.

[10] Foulkes SH Anthony EJ Group Psychotherapy: The Psycho-Analytic Approach. London: Penguin (1957).

[11] Hare AP Psychodynamics of Group Relationships. – Encyclopedia of Life Support Systems. (2002). Available from: www.eolss.net

[12] Trombini E Montebarocci O Scarponi D Baldaro B Rossi N Trombini G. Use of the drawn stories technique to evaluate psychological distress in children. Percept Mot Skills (2004) 99(3 Pt 1):975–82. 10.2466/pms.99.3.975-982 15648496

[13] Scarponi D, (a cura di). Cure Palliative Pediatriche: aspetti psico-sociali. Bentivoglio Bologna marzo: ASMEPAEdizioni (2014).

[14] Scarponi D Gruppi terapeutici per piccoli bambini. La dimensione del dire e del pensare. (Vol. 8). (2002). 7 p. Available from: www.psychiatryonline.it

[15] Scarponi D Tutto il tempo che conta. Riflessioni in PsicoOncologia Pediatrica. Bologna: Clueb Edizioni (2003).

